# Impact of Conversion from Conventional Pemafibrate to Novel Pemafibrate XR on Hypertriglyceridemia: An Observational Retrospective Study

**DOI:** 10.3390/jcm13195879

**Published:** 2024-10-02

**Authors:** Yuki Hida, Teruhiko Imamura, Koichiro Kinugawa

**Affiliations:** The Second Department of Internal Medicine, University of Toyama, 2630 Sugitani, Toyama 930-0194, Japan

**Keywords:** dyslipidemia, cardiovascular disease, pemafibrate

## Abstract

**Background:** Pemafibrate is a novel selective peroxisome proliferator-activated receptor-α modulator, which was demonstrated to reduce serum triglyceride levels with few drug-related adverse events in several clinical studies, as well as phase II and III clinical trials. One of the limitations of this medicine was the requirement of twice-daily oral administration, resulting in reduced medication adherence, particularly in elderly patients, who are rather good targets for this medicine. Recently, a once-daily extended-release (XR) tablet has been introduced. Given an improvement in medication adherence, the therapeutic efficacy of pemafibrate may be enhanced. **Methods:** Patients with hypertriglyceridemia, in whom conventional twice-daily immediate-release (IR) pemafibrate was converted to pemafibrate XR between 2023 and 2024, were eligible. Each type of tablet was prescribed for three months, respectively. A dose change was not attempted. The serum triglyceride levels were compared between 3 months pre-conversion and 3 months post-conversion using a Friedman test and a post hoc Wilcoxon signed-rank test. **Results:** A total of 46 patients were included. The median age was 62 years, and 29 were men. IR was continued for 698 (280, 1183) days before the conversion. During the last 3-month IR therapy, serum triglyceride levels remained unchanged from 171 (138, 239) mg/dL to 181 (123, 245) mg/dL (*p* = 0.78). Following the conversion, no patients had drug-related adverse events, and all patients completed 3-month XR therapy. At three months after the conversion, the serum triglyceride levels decreased significantly from 181 (123, 245) mg/dL to 146 (107, 184) mg/dL (*p* < 0.001). **Conclusions:** Pemafibrate XR might be a more promising medication than conventional IR in improving hypertriglyceridemia, probably due to improved medication adherence.

## 1. Background

Cardiovascular diseases (CVDs) remain a leading cause of morbidity and mortality worldwide, and managing dyslipidemia is a cornerstone of both primary and secondary prevention. Statins, which lower low-density lipoprotein (LDL) cholesterol, are well-established as a first-line therapy for reducing cardiovascular risk. Despite their efficacy, hypertriglyceridemia remains a significant concern as a residual risk factor [1,2]. Hypertriglyceridemia is increasingly recognized as an independent risk factor for cardiovascular events, including atherosclerosis, coronary artery disease, peripheral artery disease, and stroke. Hypertriglyceridemia is also associated with the development of small dense LDL cholesterol, which is another independent cardiovascular risk factor that has recently been focused on. Managing this residual risk has become a critical focus in lipid-lowering therapy, as many patients who achieve optimal LDL cholesterol levels with statins continue to face increased cardiovascular risk due to high triglyceride levels.

Fibrates, which activate peroxisome proliferator-activated receptor alpha (PPARα), are commonly employed to reduce triglyceride levels [3]. Nevertheless, fibrates can influence hepatic drug-metabolizing enzyme activity, thereby altering the metabolism of statins [4]. This pharmacological interaction sometimes complicates the concurrent use of fibrates and statins. This represents an unmet need, because hypertriglyceridemia often accompanies elevated LDL cholesterol levels.

Pemafibrate, a novel selective PPARα modulator, has recently been introduced as an alternative to conventional fibrates [5]. Pemafibrate has demonstrated non-inferiority in managing hypertriglyceridemia, with a lower incidence of drug-induced hepatic/renal injury compared to conventional fibrates [6,7]. A negative influence on statin is rarely observed during pemafibrate therapy, and the concomitant administration of pemafibrate and statin can be safely carried out [8]. As a result, pemafibrate has been preferred over conventional fibrates for treating hypertriglyceridemia and preventing cardiovascular diseases [9].

However, despite these advantages, pemafibrate, like other fibrates, is typically administered twice daily. This dosing frequency poses a challenge to patient adherence, particularly in those with multiple comorbidities, such as dyslipidemia, diabetes, and hypertension, which often require the concurrent use of several daily medications. Numerous studies have demonstrated that patient adherence declines as dosing complexity increases, leading to suboptimal therapeutic outcomes [10,11]. Poor adherence is particularly problematic in elderly patients, who are not only more likely to suffer from polypharmacy, but also face cognitive or physical barriers to managing their medication regimens. For these patients, simplifying therapy through once-daily dosing could significantly improve adherence and, consequently, therapeutic efficacy.

Recently, a once-daily extended-release (XR) formulation of pemafibrate was introduced, following findings of non-inferiority to conventional twice-daily immediate release (IR) in a phase III clinical trial involving carefully selected patients [12]. The XR formulation is expected to enhance medication adherence and optimize therapeutic efficacy in real-world clinical settings, in which elderly patients with multiple comorbidities receive polypharmacy [13]. In the present study, we evaluated the impact of converting from the conventional twice-daily IR formulation to the once-daily XR formulation on improving hypertriglyceridemia in real-world clinical practice. By addressing these critical aspects of treatment, this study seeks to contribute valuable insights into the real-world effectiveness of pemafibrate XR, providing a foundation for optimizing the management of hypertriglyceridemia and reducing residual cardiovascular risk in patients who have not fully responded to conventional lipid-lowering therapies.

## 2. Methods

### 2.1. Patient Selection

Consecutive patients with hypertriglyceridemia who were initially treated with the IR formulation and subsequently converted to the XR formulation were included in this retrospective study. In principle, patients were instructed to take pemafibrate IR twice-daily after taking breakfast/dinner, and pemafibrate XR once-daily after taking breakfast. Fasting time was estimated to be 12 h. Pemafibrate was generally initiated as 0.2 mg/day. In case of insufficient efficacy, the dose of pemafibrate was up-titrated to 0.4 mg/day. Throughout the treatment period, all patients received ongoing guidance on dietary and exercise modifications to manage hypertriglyceridemia. Eligible patients were those who had been on the IR formulation for more than three months and continued with the XR formulation after conversion. Excluded patients included patients who had received the IR formulation for less than three months, those who continued on the XR formulation for less than three months, those with incomplete data, and those whose pemafibrate dosage was adjusted during the observation period. Written informed consent was obtained from all participants prior to inclusion in this study. The institutional review board approved the study protocol (R2015154, 11 April 2016).

### 2.2. Study Design

The IR formulation was administered for three months prior to conversion (pre-conversion period). Following conversion, the XR formulation was continued for an additional three months (post-conversion period). Serum triglyceride levels were measured at three specific time points: (1) three months prior to conversion, (2) at the time of conversion, and (3) three months following conversion. The primary focus of this study was to compare the trends in serum triglyceride levels between the pre-conversion period (while on IR) and the post-conversion period (while on XR).

### 2.3. Biomarker Measurement

The laboratory data, including lipid parameters, were assayed by standard laboratory procedures. All serum and plasma samples were obtained at the above-stated three time points in a fasting condition and frozen at −80 degrees immediately. Small dense LDL cholesterol levels were estimated by the following formula: (Small dense LDL cholesterol) = (LDL cholesterol) − (large buoyant LDL cholesterol). Here, (large buoyant LDL cholesterol) = 1.43 × (LDL cholesterol) − [0.14 × [ln (triglyceride)] × (LDL cholesterol)] − 8.99. The ratio between triglyceride and high-density lipoprotein (HDL) cholesterol was calculated as a surrogate of small dense LDL cholesterol. Triglyceride-rich lipoprotein cholesterol was calculated as follows: total cholesterol − (LDL cholesterol and HDL cholesterol).

### 2.4. Other Clinical Data

Demographics, comorbidities, and medication data were obtained at the time of conversion as baseline characteristics. Standard laboratory data were also obtained three months before the conversion and three months after the conversion.

### 2.5. Statistical Analysis

Continuous variables were expressed as medians (with lower and upper quartiles), regardless of their distribution. Categorical variables were presented as frequencies and percentages. The trend of continuous variables over the therapeutic period was evaluated using the Friedman test and post hoc Wilcoxon signed-rank test. The trend of categorical variables over the therapeutic period was evaluated using McNemar’s test. The change in continuous variables between the pre-treatment period and post-treatment period was compared by a Mann–Whitney U test.

The association between potential baseline characteristics and the achievement of any decline in serum triglyceride levels after the conversion was investigated by logistic regression analysis. Variables with *p* < 0.05 in the univariable analysis were included in the multivariable analysis with a forced method. Here, the pre-defined potential variables at the time of conversion were as follows: age > 70 years, men, once-daily prescription, number of daily tablets, and serum triglyceride levels. The once-daily prescription was counted when patients could achieve a once-daily prescription regimen following conversion from twice-daily IR to once-daily XR (i.e., they had received all once-daily prescriptions except for twice-daily IR before conversion).

A *p*-value of less than 0.05 was considered statistically significant. All statistical analyses were conducted using SPSS Statistics version 23 (SPSS Inc., Armonk, NY, USA).

## 3. Results

### 3.1. Baseline Characteristics

A total of 50 patients who continued 3-month IR therapy and 3-month XR therapy after the conversion were included. Of them, one patient who had dose change during the therapeutic period and three patients with missing data were excluded. Finally, 46 patients were included in this retrospective study (Table 1).

The median age was 62 (52, 70) years and 29 (63%) were men. All patients had dyslipidemia and 30 (65%) had diabetes mellitus. No patients were dependent on hemodialysis. Fifteen patients had received statins and 5 patients had received ezetimibe concomitantly. IR was continued for 698 (280, 1183) days before the conversion.

### 3.2. Pemafibrate Therapy

Before the conversion, all patients continued IR therapy for over three months. Thirty-six patients received 0.2 mg/day, and the others received 0.4 mg/day. After the conversion, the dose of pemafibrate was not changed in all patients. XR was continued for three months. The dose of other lipid medications, including statins and ezetimibe, remained unchanged during the therapeutic period. The dose of medications for other comorbidities also remained unchanged during the therapeutic period. Notably, no patients received GLP-1 receptor antagonists. No patients had significant diet changes or weight loss during the therapeutic period.

### 3.3. Trajectory of Serum Triglyceride Levels

**Figure 1:**
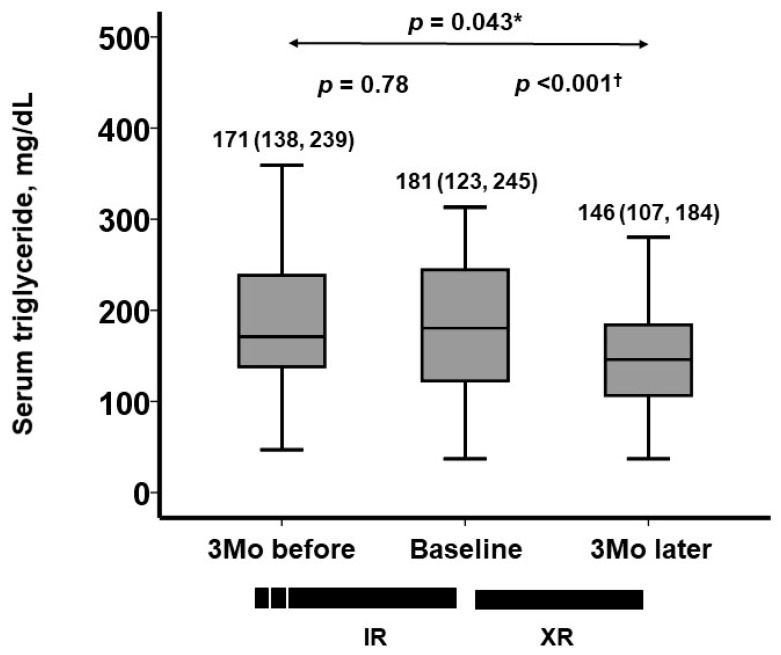
Trajectory of serum triglyceride levels during pre-conversion period (on IR treatment) and post-conversion period (on XR treatment). Serum triglyceride levels remained unchanged during pre-conversion period, whereas serum triglyceride levels decreased significantly following conversion to XR. * *p* < 0.05 according to Friedman test for trend analysis; ^†^ *p* < 0.05 according to Wilcoxon signed-rank test for post hoc two-group comparison.

**Figure 2:**
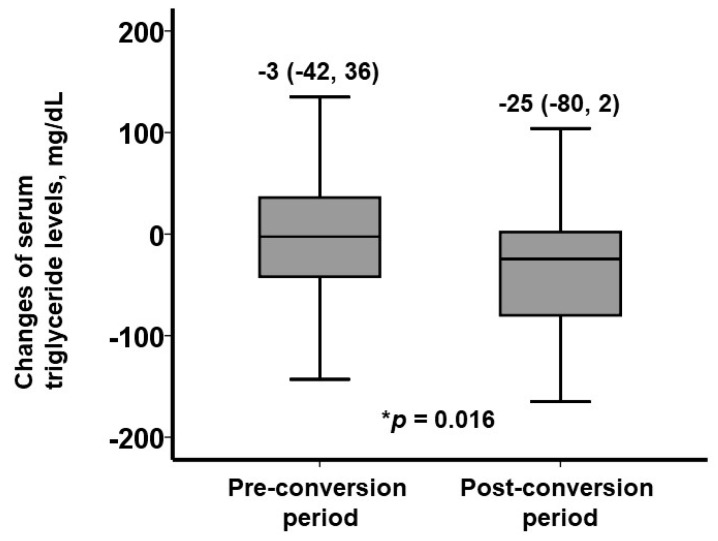
Changes in serum triglyceride levels during pre-conversion period (on IR treatment) versus post-conversion period (on XR treatment). Degree of decline in serum triglyceride levels was significantly higher during post-conversion period. * *p* < 0.05 according to Mann–Whitney U test.

**Figure 3:**
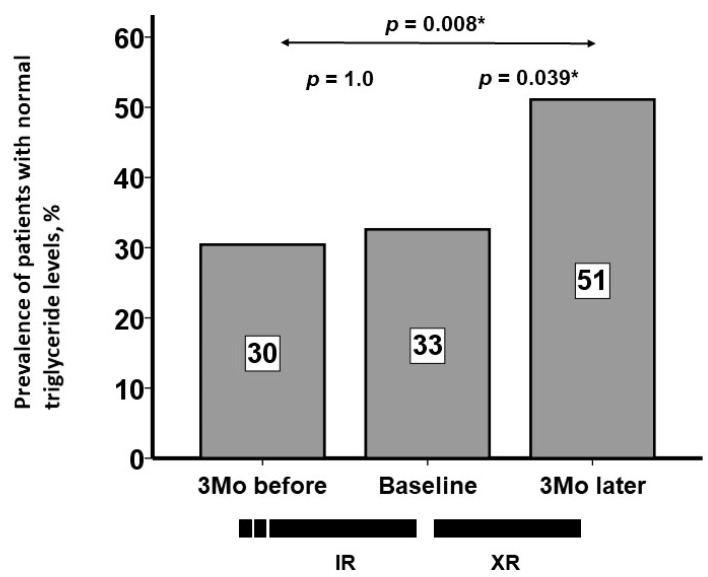
Percentages of patients who achieved serum triglyceride levels < 150 mg/dL during therapeutic period. Percentage of successful treatment (i.e., serum triglyceride levels < 150 mg/dL) remained unchanged during IR therapy but increased significantly during XR therapy. * *p* < 0.05 according to McNemar’s test.

During the 3-month pre-conversion period (on IR), serum triglyceride levels remained unchanged (*p* = 0.78; Figure 1). Following conversion to XR, serum triglyceride levels decreased significantly during the three-month XR treatment (*p* < 0.001; Figure 1). During the pre-conversion period, the median change in serum triglyceride levels was −3 (−42, 36) mg/dL. During the post-conversion period, the median change in serum triglyceride levels was −25 (−80, 2) mg/dL (*p* = 0.016 between the two therapeutic periods; Figure 2). The prevalence of patients who achieved serum triglyceride levels < 150 mg/dL (i.e., within the therapeutic range) remained around 30% during the pre-conversion period (from 30% to 33%) and increased significantly to 51% at 3 months following the conversion (*p* = 0.039; Figure 3).

### 3.4. Trajectory of Other Biomarkers

The trajectory of the other lipid parameters is displayed in Table 2. All measured lipid parameters remained unchanged during the pre-conversion period (*p* > 0.05 for all). Following conversion to XR, total cholesterol and LDL cholesterol remained unchanged (*p* > 0.05 for both). HDL-cholesterol increased significantly and triglyceride-rich lipoprotein decreased significantly (*p* < 0.001 and *p* = 0.034, respectively). The triglyceride/HDL-cholesterol ratio and estimated small dense LDL cholesterol decreased significantly (*p* = 0.001 and *p* = 0.023, respectively).

The trajectories of the other laboratory parameters are displayed in Table 3. They remained unchanged both during the pre-conversion period and post-conversion period (*p* > 0.05 for all). No patients had significant hepatic injury or acute kidney injury during the therapeutic period.

### 3.5. Factors Associated with a Decrease in Serum Triglyceride Levels Following Conversion

Potential variables were evaluated for their association with a decrease in serum triglyceride levels (i.e., improvement in hypertriglyceridemia) following conversion (Table 4). The once-daily prescription following conversion and serum triglyceride levels at the conversion remained independent predictors for the achievement of the endpoint (*p* < 0.05 for both). In other words, patients with higher serum triglyceride levels receiving once-daily prescription (except for twice-daily pemafibrate IR) are good candidates for conversion to pemafibrate XR for achieving greater improvement in hypertriglyceridemia.

## 4. Discussion

In this retrospective study, we examined the impact of switching from a twice-daily IR formulation of pemafibrate to a once-daily XR formulation in patients with hypertriglyceridemia. During the three months of IR therapy, serum triglyceride levels remained stable, whereas a significant reduction in serum triglyceride levels was observed following the conversion to XR and throughout the subsequent three months of XR therapy. No patients experienced drug-related adverse events, including hepatic injury or renal impairment. Additionally, the estimated levels of small dense LDL cholesterol and the ratio of triglycerides to HDL-cholesterol significantly decreased during the three months of XR therapy. Factors such as once-daily prescription following conversion and elevated serum triglyceride levels (insufficient control of hypertriglyceridemia) were independently associated with a further reduction in serum triglyceride levels post-conversion.

In the phase III trial, XR was shown to be non-inferior to IR in managing hypertriglyceridemia, with the percentage change in serum triglyceride levels being statistically comparable between twice-daily 0.2 mg/day IR and once-daily 0.2 mg/day XR [12]. The present study is the first to investigate the trajectory of serum triglyceride levels during IR versus XR therapy in real-world clinical practice. Notably, while serum triglyceride levels plateaued during the three-month IR therapy, they continued to decline following the conversion to XR.

Each therapeutic period in our study spanned three months, as previous findings suggested that this duration was sufficient for serum triglyceride levels to reach a plateau after the initiation of pemafibrate [14]. In the phase III trial, it was demonstrated that the therapeutic effect of 0.2 mg/day XR was equivalent to that of 0.2 mg/day IR [12]. In our study, the pemafibrate dosage remained consistent throughout the observation period.

One possible explanation for our findings is improved medication adherence. Non-adherence to medication regimens is a prevalent issue and remains a significant barrier to achieving optimal patient outcomes [15]. In general, a lower dosing frequency is associated with better adherence [16]. A previous meta-analysis suggested that reducing the dosing frequency from multiple doses to once daily improved adherence to treatment in both acutely and chronically ill patients [11]. Another prior study involving eight million insured patients with chronic obstructive pulmonary disease in the United States between 1999 and 2006 showed that once-daily dosing was linked to higher adherence compared to more frequent dosing regimens [10]. Improved adherence was correlated with reduced healthcare resource utilization and medical costs. In the present study, although direct evidence is lacking, it is possible that the twice-daily IR regimen was associated with non-adherence, leading to the suboptimal management of hypertriglyceridemia.

Furthermore, the cost of XR is lower than that of IR, and a simpler dosing regimen is associated with an improved quality of life for patients. Higher patient satisfaction may, in turn, enhance the patient–clinician relationship and further improve medication adherence.

In the present study, several factors were identified as being associated with a reduction in serum triglyceride levels after conversion, including once-daily prescription following conversion and the presence of uncontrolled hypertriglyceridemia. A complex prescription regimen is known to be linked to poor treatment adherence [17]. Patients who can enjoy a once-daily prescription when twice-daily IR is converted to once-daily XR would be particularly good candidates for the conversion. Patients at risk for non-adherence could also benefit from once-daily XR. An improvement in adherence would be particularly beneficial in patients with uncontrolled hypertriglyceridemia.

The results of this study suggest that transitioning from a twice-daily IR formulation to a once-daily XR formulation of pemafibrate may offer significant advantages in the management of hypertriglyceridemia, including improved medication adherence, enhanced patient satisfaction, and superior lipid control. However, several questions remain unanswered and warrant further investigation in future research.

First, while improved adherence is hypothesized as a key factor driving the observed reduction in serum triglyceride levels following the switch to XR, direct measurement of adherence through objective metrics, such as pharmacy refill data or electronic monitoring, would provide more conclusive evidence. Future studies should aim to assess adherence levels quantitatively and explore the extent to which adherence improvements contribute to lipid outcomes.

Second, although the present study focused on triglyceride reduction as the primary outcome and improvement in small dense LDL cholesterol as the secondary outcome, further research should examine the broader metabolic effects of XR pemafibrate, including its impact on other lipid fractions, such as VLDL and apolipoprotein B, as well as markers of inflammation and insulin sensitivity. Understanding these effects could offer a more comprehensive picture of the cardiovascular benefits conferred by XR pemafibrate. Third, long-term studies are needed to evaluate the sustained efficacy and safety of XR pemafibrate in a larger and more diverse patient population. The duration of our study was limited to three months for each treatment phase, and while this was sufficient to observe the initial effects on serum triglyceride levels, it remains unclear whether these benefits are maintained over longer periods or in more complex patient populations with comorbidities such as diabetes, obesity, or cardiovascular disease.

Finally, the potential cost-effectiveness of XR pemafibrate, considering both the lower medication cost and potential reductions in healthcare resource utilization due to improved adherence and better disease management, should be a focus of future pharmacoeconomic studies. This is particularly relevant in an era where healthcare systems are increasingly focused on optimizing outcomes while managing costs.

Our study has several important limitations that should be considered when interpreting the findings. First, the small cohort size limited the statistical power of the analysis, which may have reduced our ability to detect meaningful differences between treatment phases. As a result, all variables were treated as non-parametric data, and the lack of statistical significance in certain comparisons does not necessarily imply equivalence between groups. A larger sample size in future studies would allow for more robust statistical analyses, potentially enabling the identification of significant trends or associations that were not apparent in this study.

Second, the short-term observational period restricted our ability to assess the long-term efficacy and safety of switching from an IR to an XR formulation of pemafibrate. Although we observed notable reductions in serum triglyceride levels and other lipid markers during the three-month XR treatment phase, it remains unclear whether these effects would be sustained over an extended period. Longitudinal studies with longer follow-up periods are needed to evaluate the durability of these therapeutic benefits, as well as the potential for any late-emerging side effects or complications associated with prolonged use of the XR formulation.

Third, this study lacked a control group, which limits the strength of our conclusions. The comparisons in this study were made between pre- and post-conversion periods within the same patient population, without an external control group receiving continuous IR treatment or a placebo. This lack of control introduces the possibility of confounding factors influencing the observed outcomes, such as changes in patient behavior, diet, or other medications during the study period. Future studies with randomized, controlled designs are necessary to definitively establish the efficacy of XR pemafibrate compared to IR formulations or other therapeutic options.

Fourth, this study did not include direct measures of medication adherence, which is a key factor hypothesized to contribute to the observed improvements in triglyceride levels after conversion to XR. Although it is reasonable to infer that a once-daily dosing regimen would improve adherence relative to a twice-daily regimen, we were unable to objectively quantify adherence through methods such as pharmacy refill data or electronic monitoring. As such, the exact contribution of improved adherence to the clinical outcomes remains speculative. Future studies should incorporate methods to directly assess adherence, allowing for a clearer understanding of its impact on therapeutic efficacy.

Additionally, this study was conducted in a specific clinical setting, which may limit the generalizability of the findings to broader populations. Our cohort consisted of patients with hypertriglyceridemia who had already been receiving pemafibrate IR therapy, and the results may not be applicable to treatment-naïve patients or those with different baseline characteristics, such as more severe comorbidities. Further research in diverse populations, including those with varying degrees of lipid abnormalities and different clinical profiles, is necessary to confirm the generalizability of these results.

Finally, we were unable to investigate other potential metabolic effects of XR pemafibrate beyond serum triglyceride reduction, such as its impact on inflammation, insulin sensitivity, or cardiovascular outcomes. While triglyceride reduction is an important therapeutic target, future studies should explore the broader metabolic and cardiovascular effects of XR pemafibrate to provide a more comprehensive evaluation of its clinical benefits. Notably, the PROMINENT trial could not demonstrate a clinical impact of pemafibrate add-on therapy in patients receiving strong statin therapy on preventing cardiovascular diseases. Optimal patient selection for pemafibrate XR therapy remains a concern for future studies.

## 5. Conclusions

Pemafibrate XR may offer a more promising therapeutic alternative to conventional IR formulations in managing hypertriglyceridemia, with the potential for improved clinical outcomes. The observed advantages are likely attributable, at least in part, to enhanced medication adherence, particularly in patients who benefit from a once-daily dosing regimen following the conversion from twice-daily IR. This simplified regimen can reduce the complexity of treatment, thereby improving patient compliance, which is a critical factor in achieving optimal lipid control.

## Figures and Tables

**Table 1:** Baseline characteristics.

	*n* = 46
Age, years	62 (52, 70)
Men	29 (63%)
Body mass index	25.9 (22.2, 30.1)
Heart failure	27 (59%)
Hypertension	4 (9%)
Dyslipidemia	46 (100%)
Atrial fibrillation	3 (7%)
Diabetes mellitus	30 (65%)
Chronic kidney disease	13 (28%)
Hemodialysis	0 (0%)
Statin use	15 (33%)
Ezetimib use	5 (11%)
Anti-hypertension medication	3 (7%)
Anti-diabetic medication	28 (61%)
Anti-platelet medication	18 (39%)

Continuous variables are stated as median (25% interquartile, 75% interquartile). Categorical variables are stated as numbers (percentage).

**Table 2:** Trajectory of lipid parameters three months pre-conversion and three months post-conversion.

	3Mo before Conversion	Baseline	*p*-Value vs. 3Mo before	3Mo after Conversion	*p*-Value vs. Baseline
Total cholesterol, mg/dL	187 (154, 212)	191 (164, 219)	0.41	185 (161, 211)	0.39
LDL cholesterol, mg/dL	100 (71, 118)	97 (74, 117)	0.54	99 (82, 114)	0.75
HDL cholesterol, mg/dL	44 (34, 54)	39 (32, 51)	0.052	47 (40, 55)	<0.001 *
Triglyceride/HDL cholesterol ratio	4.2 (2.6, 5.5)	4.1 (2.9, 6.0)	0.15	2.9 (2.0, 4.8)	0.001 *
Triglyceride-rich lipoprotein, mg/dL	29.5 (26.0, 51.0)	33.5 (25.0, 51.0)	0.25	30.5 (21.0, 40.0)	0.034 *
Estimated small dense LDL cholesterol, mg/dL	38.2 (30.9, 45.8)	36.8 (31.6, 47.9)	0.27	35.8 (31.3, 42.9)	0.023 *

Pemafibrate IR was continued for over three months before the conversion. Data were obtained three months before the conversion. Pemafibrate IR was converted to pemafibrate XR at the time of baseline, when data were obtained. Following three-month pemafibrate XR treatment, data were again obtained. Continuous variables are stated as median (25% interquartile, 75% interquartile) and were compared between the groups by using a Wilcoxon signed-rank test. * *p* < 0.05. LDL, low-density lipoprotein; HDL, high-density lipoprotein.

**Table 3:** Trajectory of laboratory data three months pre-conversion and three months post-conversion.

	3Mo before Conversion	Baseline	*p*-Value vs. 3Mo before	3Mo after Conversion	*p*-Value vs. Baseline
Hemoglobin, g/dL	14.1 (13.3, 15.6)	14.0 (12.7, 15.2)	0.068	13.9 (12.5, 14.9)	0.65
Aspartate aminotransferase, IU/L	23 (13, 34)	23 (17, 36)	0.19	22 (18, 33)	0.19
Alanine aminotransferase, IU/L	21 (13, 37)	21 (11, 37)	0.93	19 (13, 33)	0.93
Gamma-glutamyl transpeptidase, IU/L	29 (17, 40)	29 (16, 36)	0.13	24 (16, 36)	0.13
eGFR, mL/min/1.73 m^2^	69.5 (57.3, 82.8)	66.9 (47.5, 78.9)	0.56	71.3 (57.4, 78.6)	0.56
Sodium, mEq/L	140 (138, 141)	140 (139, 142)	0.087	140 (139, 142)	0.067
Potassium, mEq/L	4.3 (4.0, 4.5)	4.2 (4.1, 4.4)	0.59	4.3 (4.0, 4.5)	0.59

Pemafibrate IR was continued for over three months before the conversion. Data were obtained three months before the conversion. Pemafibrate IR was converted to pemafibrate XR at the time of baseline, when data were obtained. Following three-month pemafibrate XR treatment, data were again obtained. Continuous variables are stated as median (25% interquartile, 75% interquartile) and were compared between the groups by using a Wilcoxon signed-rank test. eGFR, estimated glomerular filtration rate.

**Table 4:** The association between potential variables and a decline in serum triglyceride levels following conversion.

	Univariable Analysis		Multivariable Analysis	
	Odds Ratio (95% CI)	*p*-Value	Odds Ratio (95% CI)	*p*-Value
Age > 70 years	72.0 (0.82–62.9)	0.074		
Men	1.98 (0.57–6.79)	0.28		
Once-daily prescription	6.09 (1.17–31.6)	0.031 *	6.59 (1.03–42.2)	0.047 *
Number of daily tablets	0.87 (0.74–1.03)	0.14		
Serum triglyceride, mg/dL	1.02 (1.00–1.03)	0.011 *	1.02 (1.00–1.03)	0.014 *
Heart failure	1.63 (0.18–19.3)	0.61		
Hypertension	0.67 (0.20–2.31)	0.535		
Atrial fibrillation	1.19 (0.10–14.1)	0.89		
Diabetes mellitus	0.68 (0.19–2.46)	0.56		

All variables were obtained at the time of conversion. CI, confidence interval. * *p* < 0.05 according to logistic regression analysis for the achievement of any decline in serum triglyceride levels following conversion.

## Data Availability

Data are available upon reasonable request from the corresponding author.
